# PERK Signal-Modulated Protein Translation Promotes the Survivability of Dengue 2 Virus-Infected Mosquito Cells and Extends Viral Replication

**DOI:** 10.3390/v9090262

**Published:** 2017-09-20

**Authors:** Jiun-Nan Hou, Tien-Huang Chen, Yi-Hsuan Chiang, Jing-Yun Peng, Tsong-Han Yang, Chih-Chieh Cheng, Eny Sofiyatun, Cheng-Hsun Chiu, Chuan Chiang-Ni, Wei-June Chen

**Affiliations:** 1 Graduate Institute of Biomedical Sciences, Chang Gung University, Kwei-San, Tao-Yuan 33332, Taiwan; d0001202@stmail.cgu.edu.tw (J.-N.H.); macow911@hotmail.com (J.-Y.P.); max-fire@hotmail.com (C.-C.C.); enysofi@gmail.com (E.S.); 2 Department of Public Health and Parasitology, Chang Gung University, Kwei-San, Tao-Yuan 33332, Taiwan; Chen_th@mail.cgu.edu.tw (T.-H.C.); bluesky1986801@hotmail.com (Y.-H.C.); ice-penguin@hotmail.com (T.-H.Y.); 3 Environmental Health Department, Banjarnegara Polytechnic, Central Java 53482, Indonesia; 4 Molecular Infectious Disease Research Center, Chang Gung Memorial Hospital, Kwei-San, Tao-Yuan 33332, Taiwan; chchiu@adm.cgmh.org.tw; 5 Division of Pediatric Infectious Diseases, Department of Pediatrics, Chang Gung Children’s Hospital, Chang Gung University College of Medicine, Kwei-San, Tao-Yuan 33305, Taiwan; 6 Department of Microbiology and Immunology, Chang Gung University, Kwei-San, Tao-Yuan 33332, Taiwan

**Keywords:** dengue 2 virus, mosquito cells, PERK signaling pathway, eIF2α phosphorylation, protein translation, ER stress alleviation, cell survival

## Abstract

Survival of mosquitoes from dengue virus (DENV) infection is a prerequisite of viral transmission to the host. This study aimed to see how mosquito cells can survive the infection during prosperous replication of the virus. In C6/36 cells, global protein translation was shut down after infection by DENV type 2 (DENV2). However, it returned to a normal level when infected cells were treated with an inhibitor of the protein kinase RNA (PKR)-like ER kinase (PERK) signaling pathway. Based on a 7-Methylguanosine 5′-triphosphate (m7GTP) pull-down assay, the eukaryotic translation initiation factor 4F (eIF4F) complex was also identified in DENV2-infected cells. This suggests that most mosquito proteins are synthesized via canonical cap-dependent translation. When the PERK signal pathway was inhibited, both accumulation of reactive oxygen species and changes in the mitochondrial membrane potential increased. This suggested that ER stress response was alleviated through the PERK-mediated shutdown of global proteins in DENV2-infected C6/36 cells. In the meantime, the activities of caspases-9 and -3 and the apoptosis-related cell death rate increased in C6/36 cells with PERK inhibition. This reflected that the PERK-signaling pathway is involved in determining cell survival, presumably by reducing DENV2-induced ER stress. Looking at the PERK downstream target, α-subunit of eukaryotic initiation factor 2 (eIF2α), an increased phosphorylation status was only shown in infected C6/36 cells. This indicated that recruitment of ribosome binding to the mRNA 5′-cap structure could have been impaired in cap-dependent translation. It turned out that shutdown of cellular protein translation resulted in a pro-survival effect on mosquito cells in response to DENV2 infection. As synthesis of viral proteins was not affected by the PERK signal pathway, an alternate mode other than cap-dependent translation may be utilized. This finding provides insights into elucidating how the PERK signal pathway modulates dynamic translation of proteins and helps mosquito cells survive continuous replication of the DENV2. It was ecologically important for virus amplification in mosquitoes and transmission to humans.

## 1. Introduction

Dengue fever is caused by infection of dengue viruses (DENVs), leading to a broad spectrum of clinical symptoms including asymptomatic or undifferentiated febrile illness and sometimes severe forms of symptoms such as dengue hemorrhagic fever (DHF) and dengue shock syndrome (DSS) [1]. The virus is naturally transmitted between humans by *Aedes* mosquitoes, primarily *Aedes aegypti* [2], meaning that it is able to grow in both arthropod and human/mammalian cells. DENVs and other flaviviruses are dependent on the host endoplasm reticulum (ER) for translation, replication and packaging of their genome [3]. It results in inducing the unfolded protein response (UPR) because of the accumulation of misfolded or unfolded proteins in the ER [4]. The UPR is composed of three different classes of ER stress transducers, i.e., inositol-requiring protein-1 (IRE1), activating transcription factor-6 (ATF6) and protein kinase RNA (PKR)-like ER kinase (PERK) signaling pathways [5]. All of these consist of a luminal domain and a cytosolic domain; the luminal domain recognizes unfolded/misfolded proteins inside the ER, while the cytosolic domain relays signals to turn on downstream genes [5]. DENV infection in mammalian cells mostly leads to death, necrosis and/or apoptosis, due to the persistence of the UPR [6,7]; which has also been observed in mosquito cells [8].

Virus infection in eukaryotic cells frequently shuts down cellular protein translation due to the need to recruit ribosomes to translate viral proteins required for replication [9]. Such translational control by the virus may help cells subvert ER stress [10]. The PERK signal pathway may be an important factor as it is usually activated and subsequently phosphorylates the downstream effector, α-subunit of eukaryotic initiation factor 2 (eIF2α), which functions to suppress general protein translation [4,11]. Modulation of the UPR in DENV-infected cells was found to override the shutting down of translation, delay cell death and prolong the viral life cycle [12]. The UPR was also identified in mosquito cells with DENV2 infection [8]. However, it usually does not impair the growth of those cells [13], frequently leading to persistent infection in mosquito cells [14,15]. Regulation of the UPR via virus-controlled translation of cellular proteins could be critical for a cell’s fate during infection in both mammalian and mosquito cells.

Protein translation initiation of messenger RNA (mRNA) in cells is generally implemented by the eukaryotic translation initiation factor 4F (eIF4F) complex, which is composed of the cap-binding protein, eIF4E, the RNA helicase, eIF4A, the adaptor protein, eIF4G and other essential proteins [16]. The complex functions to link 7-methylGpppG caps at the 5′-end and poly-A tails at the 3′-end [17]. This machinery requires the binding of eIF4E to the cap structure during initiation of mRNA translation. Therefore, this process is called cap-dependent translation and is implemented by most proteins translated for physiological purposes [18]. Cap-dependent translation is initiated by activation of the mammalian target of rapamycin (TOR; mTOR) that phosphorylates eIF4E-BP in response to extracellular stimuli in such viral infections [19]. For instance, inhibiting cap-dependent translation may thus shut down protein translation of cells infected by the encephalomyocarditis virus (EMCV) or vesicular stomatitis virus (VSV) [20,21]. Based on our previous reports about mosquito cell responses to DENV2 infection [8,22], we herein aimed to demonstrate and discuss how DENV2 regulates protein translation and subsequent cell survival via the PERK signaling pathway in mosquito cells. Hypothetically, the result could be applied to elucidate how the mosquito vector can healthily transmit the DENV and maybe other arboviruses to the host.

## 2. Materials and Methods

### 2.1. Virus Propagation and Cell Culture

DENV2 (New Guinea C strain) was propagated in C6/36 cells derived from *Ae. albopictus*. Cells were cultured with minimal essential medium (MEM; GIBCO^TM^, Invitrogen, Carlsbad, CA, USA), which was supplemented with 10% fetal bovine serum (FBS) (Life Technologies, Grand Island, NY, USA), 2% non-essential amino acids, 2 g/mL Hepes (Sigma, St. Louis, MO, USA), 2.2 g/mL sodium bicarbonate (NaHCO_3_) and 0.4% antibiotic-antimycotic (GIBCO^TM^, Invitrogen) at 28 °C in a closed system without a supply of CO_2_. The propagated virus was titrated with baby hamster kidney (BHK)-21 cells (kindly provided by Chwan-Chuen King, National Taiwan University, Taipei, Taiwan), which were maintained in MEM containing 10% FBS, 2% non-essential amino acids, 2.2 g/mL NaHCO3 and 0.4% of an antimycotic (GIBCO^TM^, Invitrogen) at 37 °C in an incubator with a 5% CO2 atmosphere [22]. To inactivate the DENV2, a virus suspension was treated with a low-intensity ultraviolet (UV) source (254 nm; 800 mJ/cm^2^) for 15 min on ice in the Spectrolinker XL-1000 UV crosslinker (Spectronics, Westbury, NY, USA).

### 2.2. RNA Extraction and Reverse Transcription

The extraction of total RNA from mock- or DENV2-infected C6/36 cells using Isol-RNA Lysis Reagent (5PRIME Gaithersburg, MD, USA) followed the protocol provided by the manufacture. The extracted RNA was subsequently reversely transcribed to complementary DNA (cDNA) using the M-MLV Reverse Transcriptase (Invitrogen, Carlsbad, CA, USA). Briefly, the components including 4 μg of extracted RNA, 1 μL 100 mM Random hexamer primer (Fermentas, Glen Burnie, MD, USA) and 1 μL 10 mM dNTP Mix (Fermentas) were added into a 0.2-mL microcentrifuge tube, then filled with distilled water up to 12 μL of the total volume. The mixture was heated at 65 °C for 5 min, then 4 μL of 5× first-stand buffer, 2 μL 0.1 M DTT, 1 μL RNase OUTTM ribonucleases inhibitor (Invitrogen) and 1 μL of M-MLV reverse transcriptase were added. The final mixture was incubated at 37 °C for 60 min, followed by at 75 °C for 15 min. The formed cDNA was stored at −20 °C for further experiments.

### 2.3. Quantitative Real-Time PCR

A part of extracted cDNA was measured by a Q-PCR to quantify the expression level of specific genes. Reagents prepared for Q-PCR included forward and reverse primers (2.5 μM), 10 μL SYBR green supermix (Kapa Biosystems, Wilmington, MA, USA), 4 μL cDNA template and 5.2 μL ddH_2_O, making a final volume of 20 μL. The thermal cycles included 10 min of activation with AmpliTaq at 95 °C and 40 cycles of amplification consisting of 15 s for denaturation at 95 °C and 60 s for amplification at 60 °C, with an Applied Biosystems 7500 Real-Time PCR system (Applied Biosystems, Carlsbad, CA, USA). Primer pairs used in this experiment are listed as follows: 18S rRNA forward, 5′-ATTGACGGAAGGGCACCACCAG; 18S rRNA reverse, 5′-AGAACGGCCATGCACCACTACC (for the internal control); D2V2 forward, 5′-TGGACCGACAAAGACAGATTCTT; D2V2 reverse, 5′-CGYCCYTGCAGCATTCCAA (for DENV2).

### 2.4. Treatment of C6/36 Cells with Tunicamycin

C6/36 cells were treated with tunicamycin (Tm; EMD Millipore, Billerica, MA, USA), an ER stress inducer, to induce a strong ER stress response in mosquito cells. Briefly, 1.5 × 10^6^ C6/36 cells were seeded in a 6-cm^2^ dish and incubated at 28 °C for 24 h. Cells were subsequently treated with Dimethyl sulfoxide (DMSO) or tunicamycin at a concentration of 2, 5, 10 and 20 μg/mL for 6 h at 28 °C. Treated cells were then harvested to measure the efficiency of protein synthesis using the surface sensing of translation (SUnSET) method followed by Western blot analysis. One group of those cells further added with 5 μM PERK inhibitor was subjected to detection of eIF2, also by Western blotting.

### 2.5. Surface Sensing of Translation Used to Measure Newly Synthesized Proteins

C6/36 cells were seeded in 6-well plates with 10^6^ cells per well and incubated at 28 °C for 24 h. Except for those used as the control, cells were then infected with DENV2 at a multiplicity of infection (MOI) of 1. At various intervals of time in hours post-infection (hpi), cells were treated with 1 μM puromycin (Sigma) and incubated at 28 °C for 30 min, thus labeling nascent polypeptide chains. Cells collected from each well were then subjected to a Western blot analysis.

### 2.6. Cap Pull-Down Assay

A cap pull-down assay using immobilized γ-aminiphenyl-m^7^GTP C_10_-spacer beads (AC-155S, Jena Bioscience, Jena, Germany) was carried out to precipitate components of the intracellularly formed eIF4 complex. In brief, C6/36 cells were lysed in NP40 lysis buffer (50 mM Tris-HCL, 150 mM NaCl, 1 mM EDTA and 1% NP-40) with 1 M NaF, 200 mM Na_3_VO_4_, 1× protease inhibitor cocktail (BioShop, Burlingtom, ON, Canada) and 1× serine/threonine phosphatase inhibitor cocktail (Bionovas, Toronto, ON, Canada). About 500–1000 μg of cell lysates were incubated with 20–30 μL of beads for 6 h at 4 °C with gentle rotation. After incubation, beads were pelletized, and the supernatant was used as the run-off lysate. Pelleted beads were washed twice with NP-40 lysis buffer. Washed beads were then boiled in 50 μL of 1× sodium dodecyl sulfate (SDS) protein loading dye for 5 min. After centrifugation at 13,000 rpm for 10 min, the supernatants were stored at −20 °C for a further Western blot analysis.

### 2.7. Western Blotting

In the present study, protein profiles from C6/36 cells were analyzed by Western blotting using the corresponding antibodies listed as follows: anti-actin (clone C4) mouse monoclonal antibody (mAb; diluted 1:10^4^; EMD Millipore), anti-puromycin (12D10) mouse mAb (diluted 1:25000; EMD Millipore), anti-eIF2α rabbit polyclonal antibody (pAb; diluted 1:1000; #9722, Cell Signaling Technology, Danvers, MA, USA,), anti-phospho-eIF2α (Ser51) rabbit pAb (diluted 1:1000; #9721, Cell Signaling Technology), p70 S6 kinase (C-18) rabbit pAb (diluted 1:1000; Santa Cruz Biotechnology, sc-230, Dallas, TX, USA), phosphorylated (phospho)-*Drosophila p70* S6 kinase (Thr398) rabbit pAb (diluted 1:1000; #9209, Cell Signaling Technology), eIF4E-BP rabbit pAb (diluted 1:10^4^; produced by our lab), phospho-4E-BP (Thr37/46) rabbit mAb (diluted 1:1000; #2855, Cell Signaling Technology), eIF4E rabbit pAb (diluted 1:5000; #9742, Cell Signaling Technology), eIF4A (F52) rabbit pAb (diluted 1:5000; #2425, Cell Signaling Technology), PABP rabbit pAb (diluted 1:10^4^; #4992, Cell Signaling Technology), anti-flavivirus NS3 (diluted 1:10^4^; #YH0034, Yao-Hong Biotechnology, Taipei, Taiwan) and anti-dengue capsid mouse mAb (diluted 1:5000; kind gift from Oscar G, C Perng, National Cheng Kung University, Tainan, Taiwan). Goat anti-mouse immunoglobulin G (IgG) conjugated with a horseradish peroxidase (HRP) antibody (diluted 1:10^4^; EMD Millipore) and goat anti-rabbit IgG conjugated with an HRP antibody (diluted 1:10^4^; Sigma) were used as secondary antibodies.

Briefly, C6/36 cells were harvested at various intervals of time post-infection by centrifugation of 3000 rpm at 4 °C for 10 min. Pellets were re-suspended in 100 μL of RIPA lysis buffer consisting of 50 mM Tris-HCL, 150 mM NaCl, 1 mM EDTA, 0.25% deoxycholic acid, 1% NP-40, 0.01% Triton-X 100, 1 M NaF, 200 mM Na_3_VO_4_, 1× protease inhibitor cocktail (BioShop) and 1× serine/threonine phosphatase inhibitor cocktail (Bionovas). Subsequently, whole-cell extracts were run on 12% or 15% SDS-polyacrylamide gels, and separated proteins were electrically transferred onto polyvinylidene difluoride (PVDF) membranes with a 0.45-μm pore size (EMD Millipore). After being blocked with 5% non-fat milk in TBS-0.1% Tween 20 at room temperature for 1 h, the membrane was washed with TBS-0.1% Tween 20 and then incubated with one of the mentioned primary antibodies diluted in TBS-5% BSA at 4 °C overnight. After another wash, the membrane was incubated with a selected secondary antibody diluted in TBS-5% non-fat milk at room temperature for 1 h. After the membrane had been washed again, images were developed using Chemiluminescence Reagent (PerkinElmer^TM^ Life Science, Waltham, MA, USA) and visualized with an X-Ray Film Processor (Kodak X-OMAT 2000, KODAK, Rochester, NY, USA).

### 2.8. Treatment of C6/36 Cells with a PERK Inhibitor

Activation of PERK can be inhibited by treatment with an inhibitor (PERKi; GSK2606414) (EMD Millipore). In practice, 1.5 × 10^6^ C6/36 cells were seeded in a 6-cm^2^ dish and incubated at 28 °C for 24 h. Cells infected with DENV2 at an MOI of 1 were maintained in culture medium containing DMSO or 5 μM PERKi at indicated time points. Mock-infected cells were used as a control in the present study. Cells from each treatment were harvested at indicated time points for further experiments.

### 2.9. Analysis of the Mitochondrial Membrane Potential

The mitochondrial membrane potential (MMP) was analyzed using a MitoCapture Mitochondrial Apoptosis Detection Fluorometric kit (BioVision, Milpitas, CA, USA). In brief, cells were collected from each treatment and centrifuged at 3000 rpm for 5 min, and then, the supernatant was discarded. Pelletized cells were gently suspended by adding 1 mL of MitoCapture solution and incubated at 28 °C for 20 min in the dark. After another centrifugation at 3000 rpm for 5 min, pelleted cells were re-suspended with 1 mL of incubation buffer and analyzed with a FACScan flow cytometer (BD Biosciences, San Jose, CA, USA).

### 2.10. Measurement of Intracellular Reactive Oxygen Species

Reactive oxygen species (ROS) representing oxidative stress were measured using dihydroethidium (DHE) staining (Sigma) and 2′,7′-dichlorodihydrofluorescein diacetate (H_2_DCFDA) staining (Invitrogen) to respectively estimate the accumulation of superoxide anions and H_2_O_2_, following instructions provided by the manufacturers. Briefly, C6/36 cells from each treatment were collected and centrifuged at 3000 rpm for 5 min and then re-suspended in 1 mL of phosphate-buffered saline (PBS) containing 10 μM DHE or 10 μM H_2_DCFDA. These cells were incubated at 28 °C for 30 min in the dark, subsequently centrifuged at 3000 rpm for 5 min and then re-suspended in 1 mL of PBS. Collected cells were subjected to analysis with a FACScan flow cytometer (BD Biosciences) using red fluorescence (Channel FL1) to detect superoxide anions and green fluorescence (Channel FL2) for H_2_O_2_.

### 2.11. Assay for Measuring the Apoptosis Rate

Apoptotic cells were detected using an Annexin V-FITC/PI apoptosis detection kit (BioVision), following the manufacturer’s instructions. In brief, C6/36 cells from each treatment were collected by centrifugation at 3000 rpm for 5 min. After being gently suspended in 500 μL of binding buffer, cells were incubated with a mixture containing 5 μL Annexin V-FITC and 5 μL propidium iodide (PI) at 28 °C for 10 min in the dark. The Annexin V-FITC/PI-bound cells were ultimately analyzed with a FACScan flow cytometer (BD Biosciences).

### 2.12. Measurement of Caspase-3 and -9 Activities

Activities of caspases-3 and -9 were detected using a CaspGLOW^TM^ Fluorescein Caspase-3 staining kit (BioVision) and a CaspGLOW^TM^ Fluorescein Caspase-9 staining kit (BioVision), following the protocol from the manufacturer. Briefly, harvested C6/36 cells were centrifuged at 3000 rpm for 5 min and then re-suspended in 300 μL PBS; in which 1 μL of FITC-DEVE-FMK for caspase-3 or 1 μL of FITC-LEHD-FMK for caspase-9 was added and incubated at 28 °C for 1 h in the dark. After centrifuging at 3000 rpm for 5 min and discarding the supernatant, cells washed with and re-suspended in wash buffer were subjected to analysis with a FACScan flow cytometer (BD Biosciences).

### 2.13. PI Staining to Detect Cell Death

C6/36 cells were collected by centrifugation at 3500 rpm and 4 °C for 10 min. After the supernatant was removed, the cell pellet was fixed in ice-cold 70% ethanol in a −20 °C freezer for 1 h. The fixation solution was removed by centrifugation at 3500 rpm and 4 °C for 10 min, and then, the cell pellet was washed with PBS. These cells were treated with 0.5% Triton X-100 and 0.05% RNase A (Sigma) in PBS for 1 h at 37 °C. After a final centrifugation, pelletized cells were stained with 50 μg/mL of PI (Sigma) in PBS at 4 °C for 20 min in the dark. The cellular DNA content was measured using a FACScan flow cytometer (BD Biosciences, San Jose, CA, USA).

### 2.14. Double Luciferase Reporter Assay

The double luciferase reporter assay was used in this study to evaluate the efficiency of cap-dependent protein translation by measuring the reactions of firefly and *Renilla* luciferases in C6/36 cells. Briefly, 1.5 × 10^6^ C6/36 cells were seeded in a 6-cm^2^ dish for 24 h, followed by co-transfection with plasmids containing firefly luciferase or *Renilla* luciferase reporter genes. The transfected cells were distributed evenly in each well of a 24-well culture plate (5 × 10^5^ cells per well) for 24 h. Cells were then infected with the DENV2 (at an MOI of 1), followed by 1 h of adsorption before the addition of fresh culture medium. Cells collected at 24, 48 and 72 hpi were washed with PBS and then incubated 100 μL Passive Lysis Buffer (Promega, Madison, WI, USA) for 15 min at room temperature. After the cell lysate was centrifuged at 12,000 rpm for 30 s, a mixture of 10 μL of supernatant and 5 μL of Luciferase Measurement for Assay Reagent II (Promega) was intermediately analyzed, after 50 μL of Stop & Glo^®^ Reagent (Promega) were added to stop the reaction of firefly luciferase, with a GloMax^®^ 20/20 Luminometer (Promega).

### 2.15. Statistical Analysis

Each comparison between two means was analyzed by Student’s *t*-test at a significance level of ≤0.1, 1 or 5%. For those more than two means, a one-way analysis of variance (ANOVA) was used for the statistical test at the same level of significance.

## 3. Results

### 3.1. Shutdown of Protein Synthesis in C6/36 Cells with DENV2 Infection

**Figure 1:**
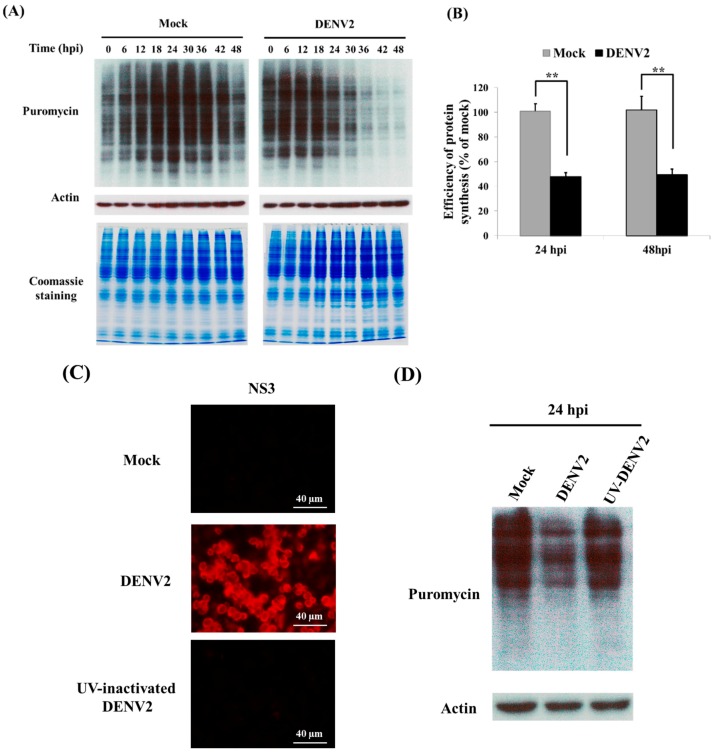

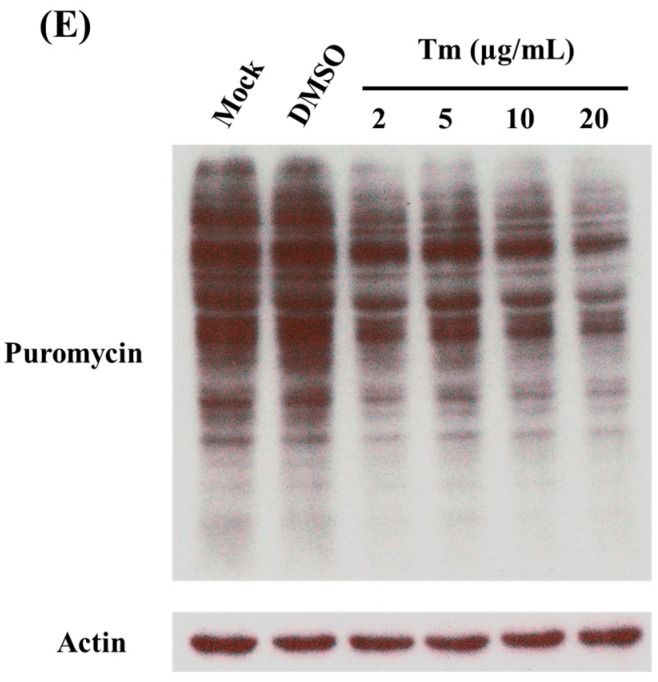
Dengue 2 virus (DENV2)-induced shutdown of total protein translation in C6/36 cells at 24 and 48 h post-infection (hpi). (**A**) Using the method of surface sensing of translation (SUnSET) to monitor and quantify global protein synthesis in infected cells. The mock- and DENV2-infected C6/36 cells were pulse-chase labelled with puromycin for 30 min before cells were harvested. Protein synthesis rate was analyzed by Western blot with an antibody against puromycin. Western blot analysis of actin and Coomassie Blue staining of total cellular protein were included to prove equal protein loading. Results showed that the total protein amount had been reduced by 24 hpi, and this persisted to 48 hpi; (**B**) Histograms represent the quantification of the intensities of puromycin-incorporated bands from three independent experiments. Changes between virus- and mock-infected cells significantly differed at both time points (Student’s *t*-test; ** *p* < 0.01); (**C**) The viral NS3 protein was only detected in cells infected by infectious DENV2, not in those inoculated with a UV-inactivated virus according to the results of the immunofluorescent assay; (**D**) The method SUnSET also showed that total protein synthesis decreased in cells infected with untreated DENV2, but there was no obvious change in cells inoculated with a UV-inactivated virus; (**E**) In C6/36 cells treated with tunicamycin (Tm), a strong endoplasmic reticular (ER) stress inducer, for 6 h, the expression of total proteins decreased, even when the concentration of the drug was as low as 2 µg/mL.

SUnSET is a nonradioactive fluorescence-activated cell sorting-based assay that allows the monitoring and quantification of global protein synthesis in cells [23]. Herein, the total protein amount in C6/36 cells with DENV2 infection was shown to be reduced at 24 hpi, and this persisted to 48 hpi (Figure 1A); changes between infected and uninfected cells at both time points significantly differed (Student *t*-test; ** *p* < 0.01) (Figure 1B). The viral NS3 protein can only be detected in cells inoculated with infectious DENV2, but not the virus inactivated by treatment with UV light (Figure 1C). Furthermore, protein shutdown was not shown in cells inoculated with UV-inactivated DENV2 for 24 h, while it was observed in untreated DENV2 (Figure 1D). In the meantime, total proteins were obviously reduced in cells treated with different concentrations of tunicamycin (Tm), which is known as a strong ER stress inducer (Figure 1E). This reveals that shutdown of global protein translation triggered by DENV2 entering C6/36 cells was compatible to that treated with the ER stress inducer.

### 3.2. Cap-Dependent Protein Translation in DENV2-Infected C6/36 Cells

**Figure 2:**
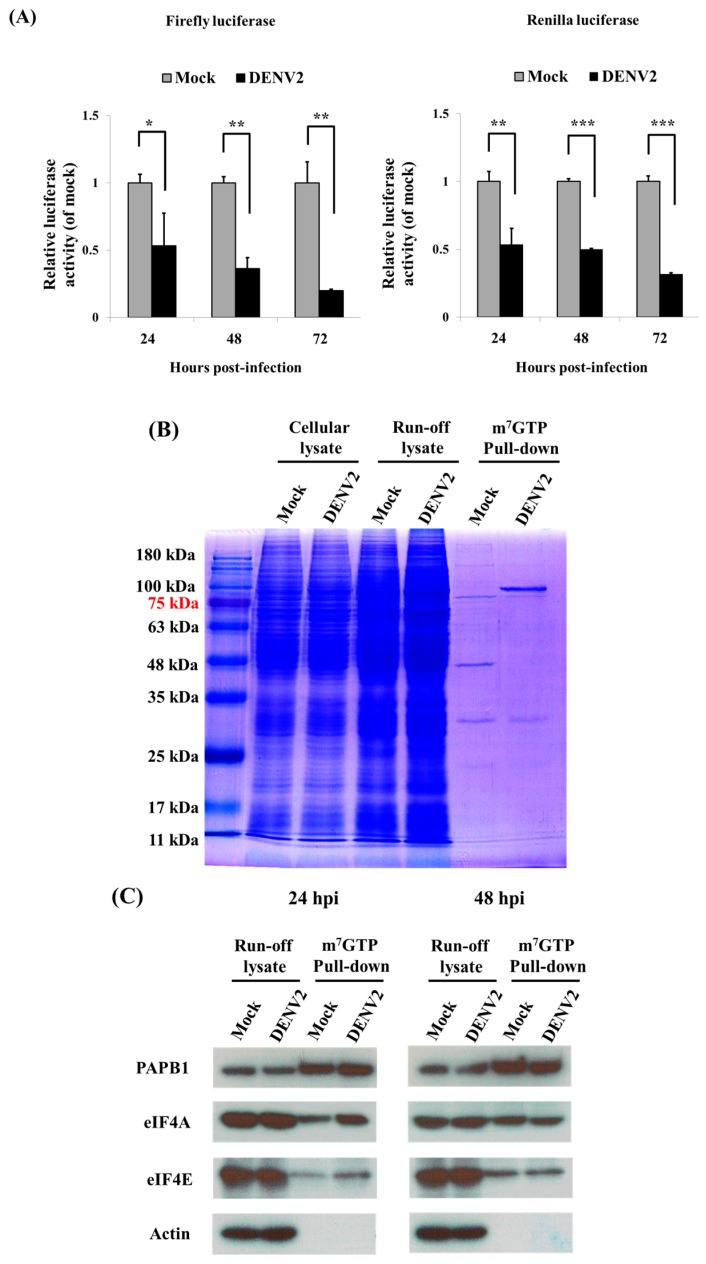
Demonstration of cap-dependent protein translation in dengue 2 virus (DENV2)-infected C6/36 cells. (**A**) A double luciferase detection system with transfection of two plasmids containing firefly luciferase or *Renilla luciferase* was used to evaluate cap-dependent protein translation in C6/36 cells; both firefly and *Renilla* luciferases exhibited significantly reduced activities in DENV2-infected at 24, 48 and 72 h post-infection (hpi) compared to cells with mock-infection (Student’s *t*-test; * *p* < 0.05, ** *p* < 0.01, *** *p* < 0.001); (**B**) With an m7GTP pull-down assay followed by SDS-PAGE, only specific proteins were left and are shown on the gel; (**C**) Detection by Western blotting with specific antibodies, proteins, including eIF4E, eIF4A and poly-A binding protein (PABP), were identified without significant difference in both mock- and DENV2-infected cells after pull-down by m7GTP in addition to that found in run-off cell lysate both at 24 and 48 hpi. This indicated that an eIF4F complex that served as the cap-dependent protein translation machinery had been formed.

Cellular proteins were generally synthesized via cap-dependent protein translation, which was evaluated in DENV2-infected C6/36 cells by transfection with a dual luciferase detection system. The system utilizes a plasmid containing firefly and *Renilla* luciferases, the promoter of which triggers the translation of both reporter proteins in a cap-dependent manner. Results showed that both firefly and *Renilla* luciferases had significantly reduced activities in C6/36 cells with DENV2 infection at 24, 48 and 72 hpi compared to the relative luciferase activities of mock-infected cells (Student’s *t*-test; * *p* < 0.05, ** *p* < 0.01, *** *p* < 0.001) (Figure 2A). With the m7GTP pull-down assay followed by SDS-PAGE and Coomassie Blue staining, only a few proteins were shown, representing specific proteins that had been pulled down (Figure 2B). By detection with Western blot analysis using specific antibodies, components of the eIF4F complex, including eIF4E, eIF4A and PABP, were indifferently identified in both mock- and DENV2-infected cells after pull-down with m7GTP, especially in cells with DENV2 infection for 24 h (Figure 2C). This indicates that an eIF4F complex serving as the cap-dependent protein translation machinery had been formed; however, DENV2 infection may have interfered with subsequent protein translation.

### 3.3. Activation of the TOR Signaling Pathway in C6/36 Cells Infected with the DENV2

**Figure 3:**
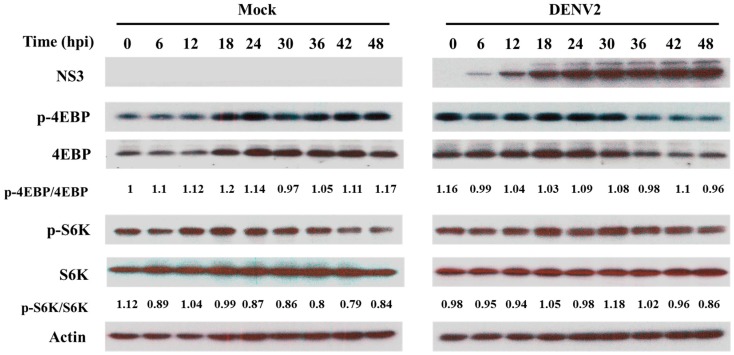
Activation of the target of rapamycin (TOR) signaling pathway by the dengue 2 virus (DENV2) in C6/36 cells. According to the Western blotting analysis carried out in DENV2-infected C6/36 cells, eIF4E-BP was shown to be phosphorylated at a very early stage of infection by the DENV2 (<12 h post-infection (hpi)) and was at a lower level at a later stage of infection (>36 hpi), compatible with a reduction in eIF4E-BP expression. According to the ratio of phosphorylation/total eIF4E-BP, its change of phosphorylated level may be dependent on the expression of eIF4E-BP. One of TOR’s downstream genes, S6 kinase (S6K), was usually phosphorylated particularly at 18 hpi in C6/36 cells, even though its protein level remained at a constant level throughout the same period of infection. In the meantime, NS3 viral proteins were persistently expressed throughout the 48-h infection period. The level of actin was included to prove equal loading of protein in each line. This reveals that the TOR signaling pathway may have been activated and involved in controlling cap-dependent translation of cellular proteins.

Since regulation of cap-dependent translation has been related to TOR signaling [24], to access the activation of the TOR signaling pathway, its downstream target gene eIF4E-BP was detected due to the absence of an appropriate antibody for recognizing TOR activation in mosquito cells. The result showed that eIF4E-BP was phosphorylated at a very early stage of infection by DENV2 (<12 hpi); however, it was at a lower level in a later stage of infection (>36 hpi). In spite of this, such changes may depend on the expressed protein according to the ratios between the phosphorylated form and the amount of eIF4E-BP (Figure 3). Another of TOR’s downstream genes S6K was also shown to be phosphorylated, as shown in mammalian cells infected by West Nile virus [25], particularly in a later stage of infection, e.g., 30 hpi in C6/36 cells, although its protein expression level remained at a constant level throughout the same period of infection (Figure 3). Results revealed that the TOR signaling pathway may have retained its activity involved in controlling cap-dependent protein translation in C6/36 cells infected by the DENV2. This suggests that most cellular proteins are synthesized principally via cap-dependent translation in C6/36 cells. Since NS3 viral proteins were persistently expressed throughout the period of infection for 48 h in DENV2-infected C6/36 cells (Figure 3), viral proteins may be translated by an alternate mode of translation disregarding the TOR signaling pathway [26].

### 3.4. Recovery of Cellular Protein Synthesis in DENV2-Infected C6/36 Cells Treated with a PERK Inhibitor

**Figure 4:**
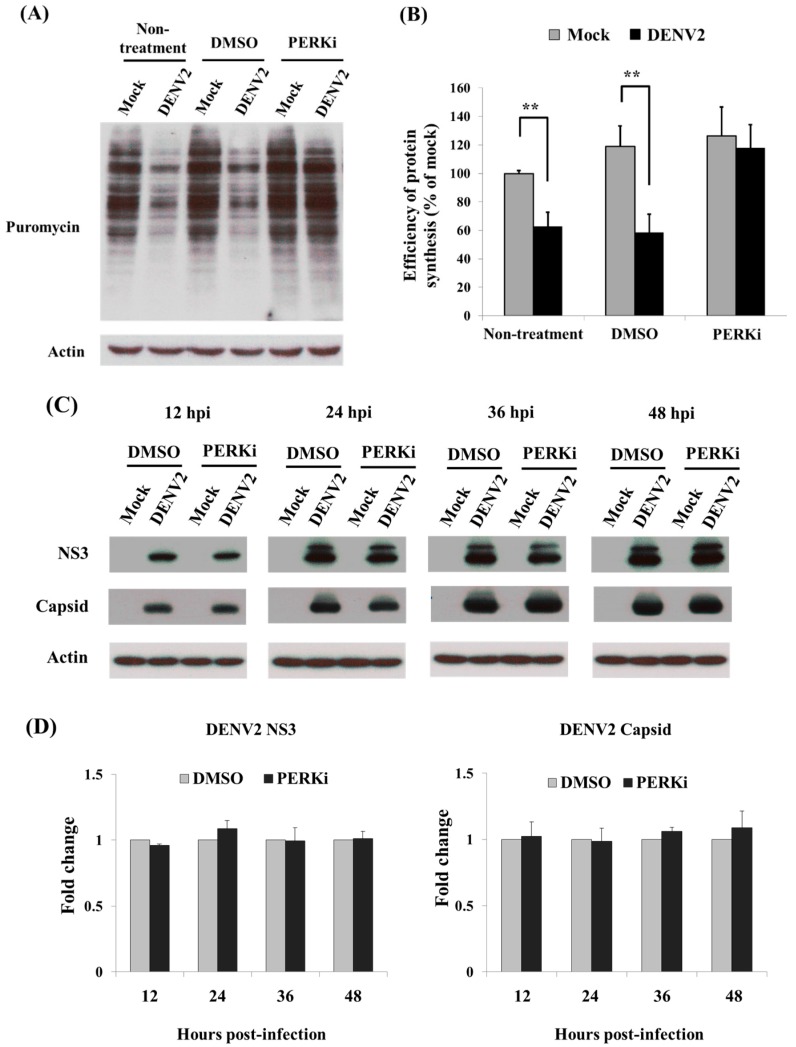

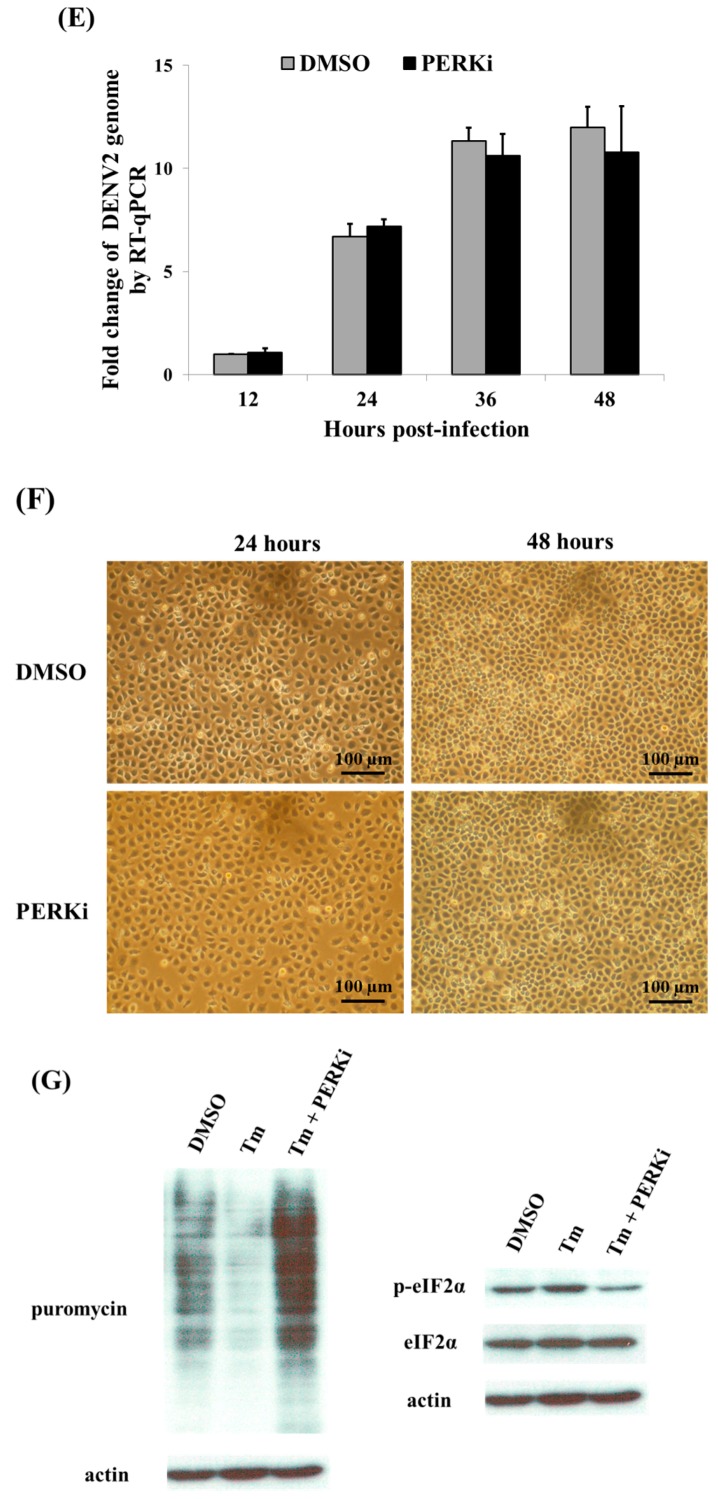
Cellular protein synthesis was recovered in dengue 2 virus (DENV2)-infected C6/36 cells after being treated with a PERK inhibitor. (**A**) After DENV2-infected C6/36 cells (MOI = 1) were treated with a PERK inhibitor (PERKi; GSK2606414) at 5 μM, DENV2-induced inhibition of protein translation obviously recovered based on results from the method of SUnSET; (**B**) Histograms represented quantitated intensities of puromycin-incorporated proteins from three independent experiments of C6/36 cells. The difference of protein translation levels between the two groups of PERKi-treated cells with and without infected by DENV2 was shown to be not significant (Student’s t-test; *p* > 0.05). In the two control groups (non-treatment and DMSO only), protein translation levels were significantly different (** *p* < 0.01); (**C**) Looking at capsid and NS3 of dengue proteins by Western blotting analysis, both of them are continuously synthesized in C6/36 cells treated with PERK inhibitor even though the cells have been infected by DENV2 for 48 h; (**D**) Quantitatively, protein amounts of these two viral protein did not show significant differences in cells treated with PERK inhibitor or not; (**E**) For viral RNA levels detected by quantitative real-time RT-PCR, it did not show significantly differences between C6/36 cells with and without PERK inhibitor treatment, either; (**F**) Upon observation on the effect of the PERK inhibitor on C6/36 cells in the experiment, no obvious morphological defects were observed in cells treated with the same concentration of the inhibitor for up to 48 h, indicating that the PERK inhibitor did not cause adverse effects on cells; (**G**) By the method of SUnSET, reduced protein translation in cells treated with tunicamycin (Tm) was also recovered by adding the PERK inhibitor (5 μM). The phosphorylated eIF2α elicited by treatment with Tm turned out to be a lower level of amount according to the Western blotting analysis. It reflected that the PERK signaling pathway regulates cellular protein translation after endoplasmic reticular stress was induced in C6/36 cells.

In order to check the role of the PERK signaling pathway in cellular protein synthesis, a PERK inhibitor (GSK2606414) at a concentration of 5 μM was used to treat DENV2-infected C6/36 cells. Though off-target-effects have ever been of concern, this drug seems to be specific enough for observation on its functional trend of PERK inhibition [27,28]. The result showed that inhibition of total protein translation was recovered (Figure 4A). Statistically, it returned to a level close to normal, showing an insignificant difference between PERKi-treated cells with and without infected by DENV2 (Student’s *t*-test; *p* > 0.05) (Figure 4B). Dengue viral proteins, capsid (C) and NS3, remained to be synthesized in PERK inhibitor-treated C6/36 cells even those that have been infected by DENV2 for 48 h (Figure 4C). No significant difference of protein amounts was shown between cells with and without PERK inhibitor treatment (Figure 4D). Meanwhile, no significant difference of viral RNA level was shown between cells with and without PERK inhibitor treatment (Figure 4E). In addition, no obvious morphological defects were observed in cells treated with the inhibitor for up to 48 h (Figure 4F), indicating that at 5 μM, the PERK inhibitor did not have adverse effects on cells. Moreover, reduced protein translation in cells treated with tunicamycin (Tm) was recovered by adding the PERK inhibitor at 5 μM (Figure 4G). In the meantime, phosphorylated (p-) eIF2α was shown to increase after treatment with Tm, but returned to be a lower level of amount when the PERK inhibitor was added (Figure 4G). The eIF2α is known to be a key downstream target of the PERK signaling pathway and involved in protein translation in eukaryotic cells [29]. This suggested that the PERK signaling pathway is involved in regulating cellular protein translation when ER stress is induced by DENV2 in mosquito cells.

### 3.5. Evaluation of ER Stress by Measuring the MMP

**Figure 5:**
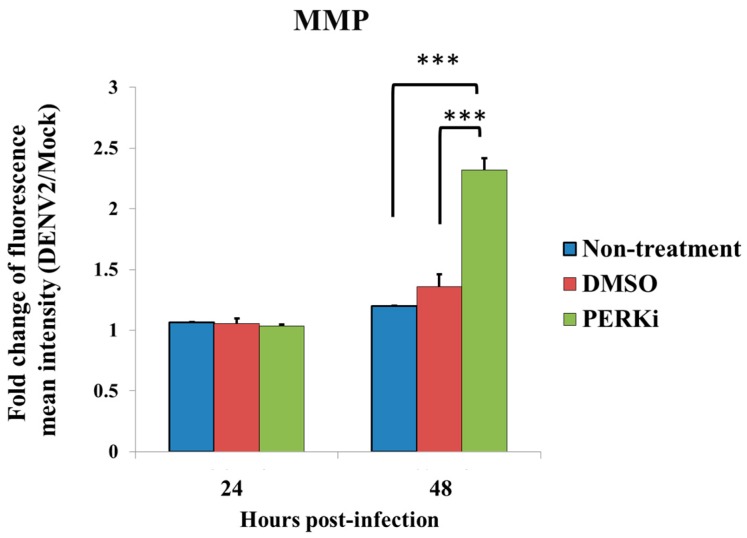
Evaluation of endoplasmic reticular (ER) stress by measuring the mitochondrial membrane potential (MMP) in dengue 2 virus (DENV2)-infected C6/36 cells. To measure MMP, detection of the FITC channel for green MitoCapture monomers by FACScan flow cytometry was carried out in DENV2-infected C6/36 cells (MOI = 1) with and without PERK inhibitor (5 μM) treatment. The MMP in the group treated with the PERK inhibitor had significantly increased at 48 h post-infection (hpi) (a 2.32-fold increase) compared to cells in the two control groups (1.20-fold for the untreated and 1.36-fold increases for the DMSO-treated group) (Student’s *t*-test; *** *p* < 0.001).

The MMP generally changes due to a transition in mitochondrial permeability, which has been demonstrated to change in C6/36 cells, as well as in mammalian BHK-21 cells infected by DENV2 for 24 h [8]. To evaluate the MMP in this study, FACScan flow cytometry was used to measure the Fluorescein isothiocyanate (FITC) channel for green MitoCapture monomers in mock- and DENV2-infected C6/36 cells with and without treatment with the PERK inhibitor. Results revealed a difference in the induction of the MMP between cells in the two groups, particularly at 48 hpi. Quantitatively, the fluorescent intensity of DENV2-infected C6/36 cells treated with the PERK inhibitor at 48 hpi (2.32-fold) significantly increased compared to the two control groups (by 1.2-fold compared to untreated cells and 1.36-fold compared to DMSO-treated cells) (Student’s *t*-test; *** *p* < 0.001), while no statistical difference was shown at 24 hpi (Figure 5). This indicates that a change in the MMP is one of the essential steps possibly involved in the PERK signaling pathway. In fact, it is an important step in the process of inducing apoptosis in cells under ER stress [30].

### 3.6. Involvement of the PERK Signaling Pathway in Generating ROS in C6/36 Cells with DENV2 Infection

**Figure 6:**
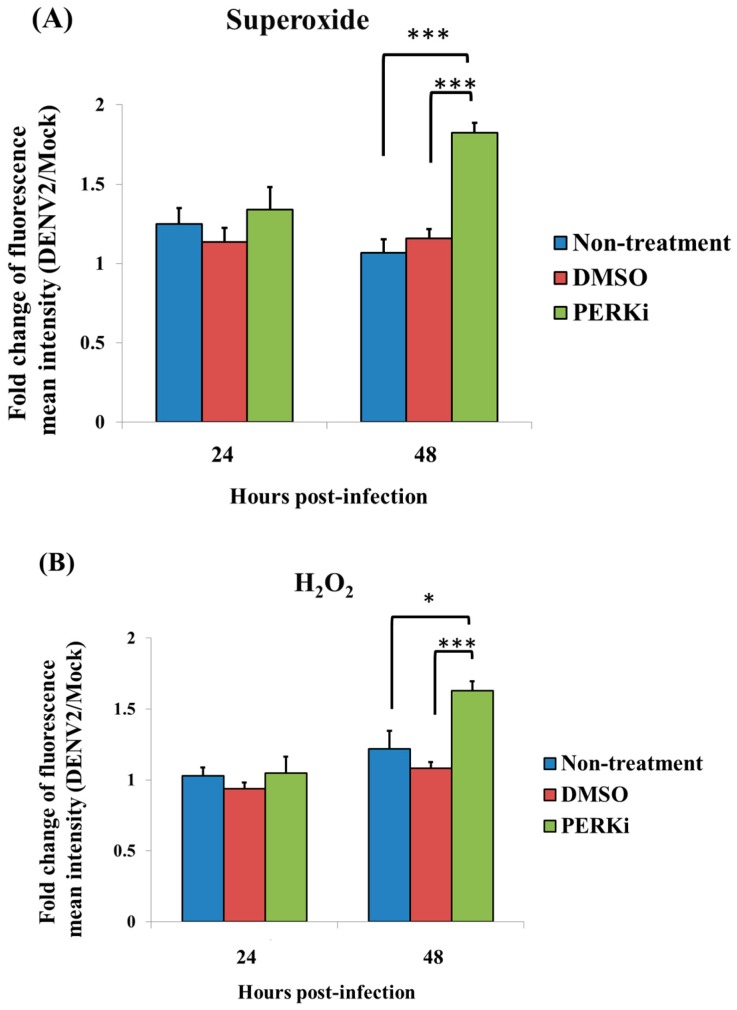
The PERK signaling pathway is involved in generating reactive oxygen species (ROS) accumulation in C6/36 cells with dengue 2 virus (DENV2) infection. Superoxide anions (O^2−^) are one of the main ROS and are usually converted to hydrogen peroxide (H_2_O_2_). (**A**) Using FACScan flow cytometry to detect the relative fluorescent intensity, superoxide anions exhibited no obvious change in cells with DENV2 infection (MOI = 1) at 24 h post-infection (hpi; 1.34-fold) even though a PERK inhibitor (5 μM) was applied. A significant (1.82-fold) increase in superoxide anions was measured at 48 hpi, compared to those in the control groups (1.07-fold for the untreated and 1.16-fold for the DMSO-treated group; Student’s *t*-test; *** *p* < 0.001); (**B**) There was a similar changing trend in hydrogen peroxide in the same cell groups, leading to a significant increase at 48 hpi (1.63-fold; Student’s *t*-test; * *p* < 0.05, *** *p* < 0.001), but not at 24 hpi, (1.05-fold).

Superoxide anions (O^2-^) are one of the main ROS and are usually converted to hydrogen peroxide (H_2_O_2_); both of these are free radicals that may accumulate and induce the ER stress in the cell [31]. Using FACScan flow cytometry, the relative fluorescent intensity of superoxide anions detected in C6/36 cells with DENV2 infection for 24 h did not significantly change (1.34-fold) even when a PERK inhibitor was applied (Figure 6A). Nevertheless, it had significantly increased by 48 hpi (a 1.82-fold increase) compared to cells in the control groups (1.07- and 1.16-fold for untreated and DMSO-treated cells, respectively) (Student’s *t*-test; *** *p* < 0.001). A similar changing trend of hydrogen peroxide was shown in the same cell groups, leading to a significant increase at 48 hpi (1.63-fold; Student’s *t*-test; * *p* < 0.05, *** *p*<0.001), but not at 24 hpi (1.05-fold) (Figure 6B). This reflects that the PERK signaling pathway may be involved in changing the accumulation of ROS in C6/36 cells with DENV2 infection.

### 3.7. The PERK Signaling Pathway Is Involved in Reducing Cell Death of C6/36 Cells Infected with the DENV2

**Figure 7:**
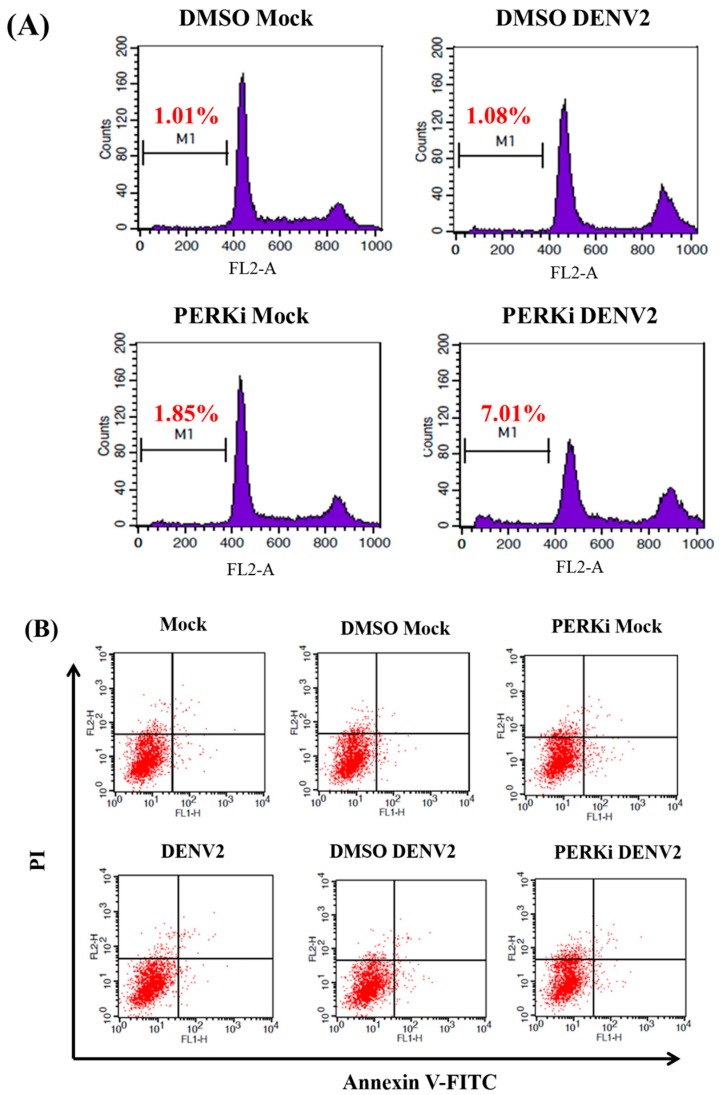

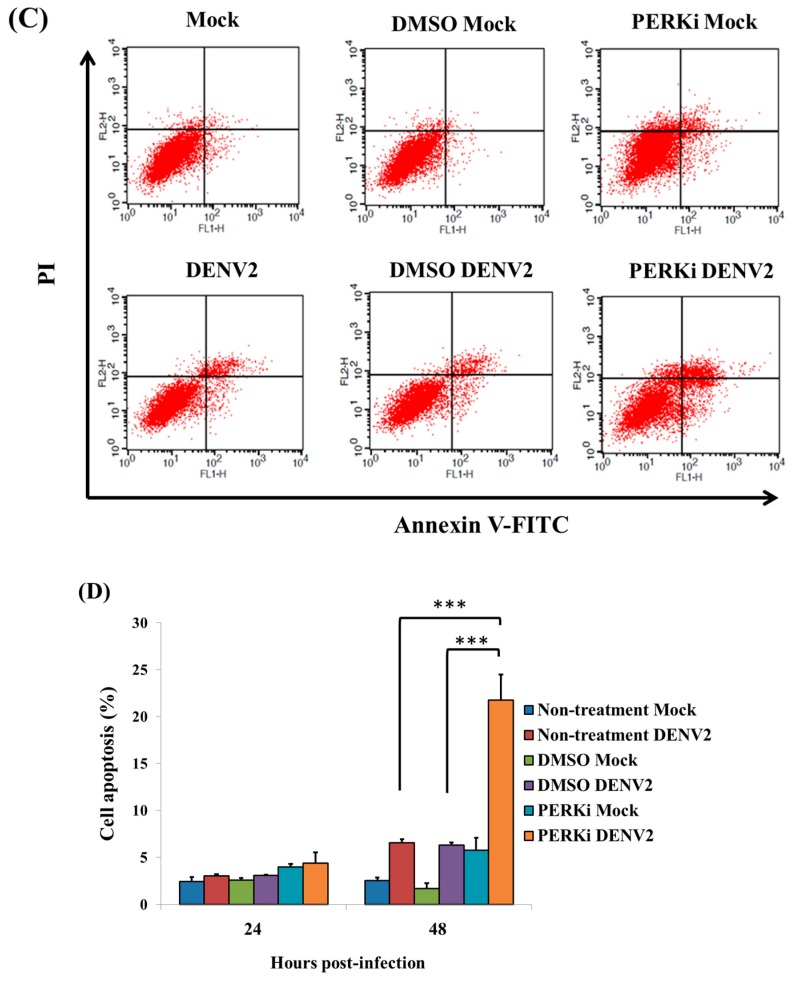
The involvement of the PERK signaling pathway in a reduction of cell death in C6/36 cells with dengue 2 virus (DENV2) infection. (**A**) Cell cycle progression was detected by PI staining in DENV2-infected C6/36 cells (MOI = 1) treated with a PERK inhibitor (5 μM). The result revealed that there was a higher rate (7.01%) of the sub-G_1_ phase at 48 h post-infection (hpi); (**B**) Staining of DENV2-infected cells with Annexin V-FITC/PI followed by fluorescence activated cell sorting (FACS) revealed that slight apoptosis-related cell death had occurred at 24 hpi; (**C**) Apoptosis-related cell death in cells of the same group as above had obviously increased by 48 hpi; (**D**) At 48 hpi, there was a statistically higher apoptosis-related cell death rate in the group treated with the PERK inhibitor and infected with the DENV2 compared to the other two control groups with DENV2 infection (Student’s *t*-test; *** *p* < 0.001).

**Figure 8:**
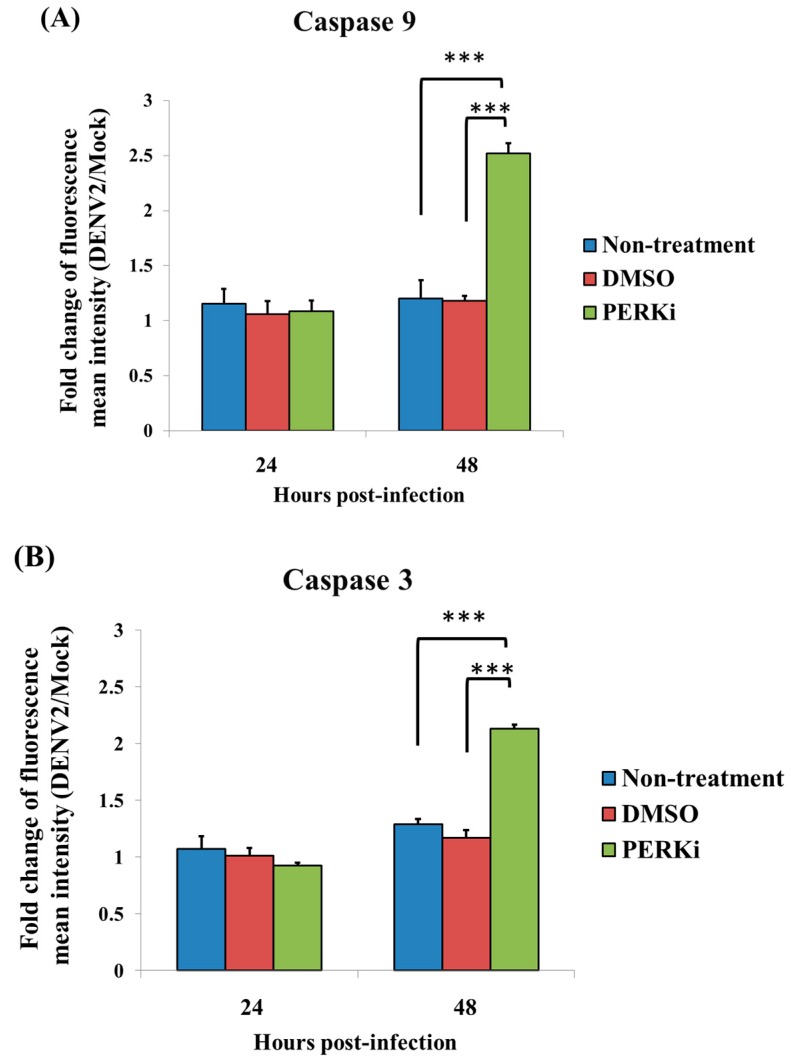
The effect of the PERK inhibitor on activation of caspases-9 (the initiator) and -3 (the effector) in C6/36 cells with dengue 2 virus (DENV2) infection by using FACScan flow cytometry. (**A**) In DENV2-infected C6/36 cells (MOI = 1), no evident change in caspase-9 had occurred in cells by 24 h post-infection (hpi), even when they were treated with a PERK inhibitor (5 μM). However, it had increased by 48 hpi (2.52-fold increase) compared to the two control groups (1.20- and 1.18-fold for untreated and DMSO-treated cells, respectively; Student’s *t*-test; *** *p* < 0.001); (**B**) A similar changing trend of caspase-3 was seen in the same cell group, having remained unchanged at 24 hpi, but having significantly increased at 48 hpi (2.13-fold increase) compared to the control groups (1.29-fold for the untreated and 1.17-fold for the DMSO-treated group; Student’s *t*-test; *** *p* < 0.001). Very likely, the DENV2-induced PERK signaling pathway was important for C6/36 cells avoiding induction of apoptosis during infection.

The sub-G1 phase corresponds to cells with fragmented DNA genome, thus representing the cells undergoing apoptosis during the cell cycle. In this study, the cell cycle progression was detected by PI staining in DENV2-infected C6/36 cells treated with a PERK inhibitor, revealing that there was a higher rate (7.01% at 48 hpi) of cells in the sub-G_1_ phase (Figure 7A). When DENV2-infected cells were stained with Annexin V-FITC/PI, slight apoptosis-related cell death was found to have occurred at 24 hpi (Figure 7B), but it had significantly increased by 48 hpi (Figure 7C). There was a statistically-significant difference between cells treated with the PERK inhibitor followed by DENV2 infection compared to the other two control groups with infection (Student’s *t*-test; *** *p* < 0.001) (Figure 7D). Activation of caspases-9 (the initiator) and -3 (the effector), which leads to apoptosis, was previously demonstrated in C6/36 cells with DENV2 infection [22]. In this study, we further assessed its occurrence in DENV2-infected C6/36 cells with DENV2 infection. Results showed that no evident change in caspase-9 had occurred in cells by 24 hpi, even when they had been treated with the PERK inhibitor, while it had obviously increased at 48 pi (a 2.52-fold increase) compared to the two control groups (1.20-fold for untreated and 1.18-fold for DMSO-treated cells) (Student’s *t*-test; *** *p* < 0.001) (Figure 8A). A similar changing trend of caspase-3, remaining unchanged at 24 hpi, but significantly increased at 48 hpi (a 2.13-fold increase), was shown compared to the control groups (1.29-fold for untreated and 1.17-fold for DMSO-treated cells) (Student’s *t*-test; *** *p* < 0.001) (Figure 8B). This suggests that the DENV2-induced PERK signaling pathway plays an important role in C6/36 cells, allowing them to avoid the induction of apoptosis in response to infection.

### 3.8. Activity of eIF2α in the PERK Signaling Pathway during Protein Translation of C6/36 Cells with DENV2 Infection

**Figure 9:**
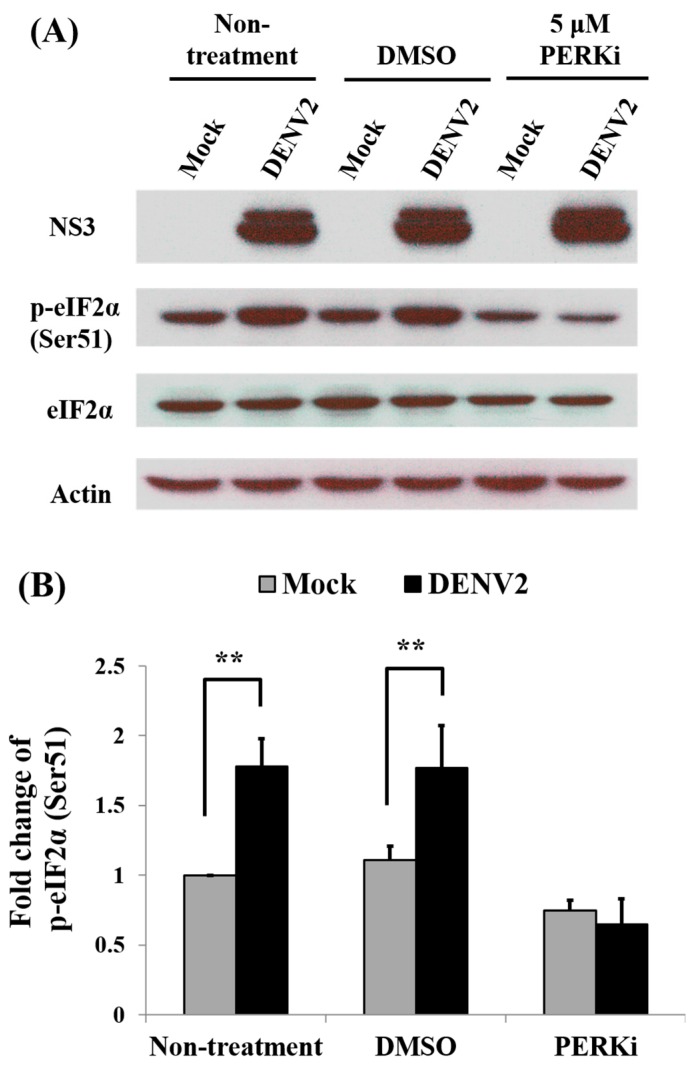
Activity of eIF2α in the PERK signaling pathway during protein synthesis in dengue 2 virus (DENV2)-infected C6/36 cells. (**A**) The C6/36 cells were infected with DENV2 at MOI of one, then treated with 5 μM PERK inhibitor or DMSO. The activity of eIF2α was shown to increase the degree of phosphorylation in response to viral infection, while its protein level mostly remained at a stable level. On the other hand, the amount of phosphorylated eIF2α was significantly reduced when the PERK inhibitor was applied to infected cells. As synthesis of the NS3 viral protein did not show an evident change even when cells had been infected for 24 h, an alternative mechanism for translation of viral proteins in C6/36 cells may exist; (**B**) Statistically, levels of phosphorylated eIF2α significantly increased (Student’s *t*-test; ** *p* < 0.01) in cells without PERK inhibitor treatment and in those cells treated with DMSO only. In contrast, there was no significant difference between DENV2-infected cells and the control groups (Student’s *t*-test; *p* > 0.05). It revealed that the PERK signaling pathway may have been important during protein synthesis in DENV2-infected mosquito cells.

As mentioned above, eIF2α is involved in protein translation in eukaryotic cells via the PERK signaling pathway. Since that are no available tools for directly detecting PERK activation of insect cells in hand, measuring phosphorylation of eIF2α was used as the alternative way showing PERK activation in this study. When observing its activity, an increased degree of phosphorylation in response to viral infection was detected, while its protein amount remained stable. Importantly, phosphorylated eIF2α was significantly reduced when the PERK inhibitor was applied to infected cells (Figure 9A). This indicates that phosphorylation of eIF2α triggered by DENV2 infection is deeply involved in the translation of cellular proteins through the PERK signaling pathway in C6/36 cells. Quantitatively, the level of phosphorylated eIF2α significantly increased (Student’s *t*-test; ** *p* < 0.01) in DENV2-infected cells without PERK inhibitor treatment and in those treated with DMSO only. In contrast, no evident change was shown in virus-infected cells treated with a PERK inhibitor (Student’s *t*-test; *p* > 0.05) (Figure 9B). In the meantime, synthesis of the NS3 viral protein did not show evident change even in the cells that were infected for 24 h. This suggested that translation of viral proteins may not utilize a canonical cap-dependent translation mechanism in C6/36 cells.

## 4. Discussion

Translation of global cellular proteins in this study was shown to decrease in response to DENV2 infection. A similar reaction was also observed in mammalian cells infected with lytic RNA and DNA viruses [32,33]. This suggests that arboviral infections in host cells, either mammal or mosquito derived, change the translation of cellular proteins [34]. Within cells, proteins are generally synthesized using mRNA as a template; this has been highly conserved across species of eukaryotes throughout evolution [35]. It usually follows a cap-dependent translation mode [18]; which is generally regulated by activated mTOR, which contributes to the phosphorylation and activation of eIF4E-BP and the ribosomal protein, S6 kinase (S6K) [36]. A TOR-mediated pathway may have been involved in such cap-dependent translation, which may be inhibited when dephosphorylated eIF4E-BP binds to eIF4E and thus prevents the formation of the eIF4F complex [24]. This is compatible with our observations in DENV2-infected C6/36 cells, indicating that global cellular proteins are translated by a cap-dependent translation mode [37]. Normally, they are processed by the 40S ribosomal subunit that binds in the vicinity of the cap structure at the 5′-end of mRNA and scans until an AUG codon in the mRNA is encountered [18,38].

Components of the eIF4F complex, including eIF4E, eIF4A and PABP, were identified according to the m7GTP pull-down assay performed in this study. The results firmly revealed that canonical cap-dependent protein translation occurred in mosquito cells even when they were infected by the DENV2, as the formation of the eIF4F complex was obviously observed in C6/36 cells. The dual luciferase reporter assay in this study also supported that DENV2 infection negatively regulates cap-dependent translation in C6/36 cells. eIF2α being obviously phosphorylated in C6/36 cells with DENV2 infection firmly demonstrates that Met-tRNAi binding to the 40S ribosome may have been impaired, even though the eIF4F complex eventually formed [39,40].

The UPR as mentioned above is caused by the accumulation of unfolded or misfolded proteins in the ER; which actually resulted in changes in X-box binding protein 1 (XBP1) and splicing activity [41]. The UPR generally leads to activation of three signaling pathways including PERK, IRE1 and ATF6 [5,42]. Apparently, the PERK signaling pathway is highly involved in modulating the dynamic translation of cellular proteins and subsequently regulating ER stress induced by DENV2 infection. Interestingly, decreased translation of global cellular proteins in DENV2-infected C6/36 cells significantly recovered once a PERK inhibitor (GSK2606414) was applied to cells. This indicates that the PERK signaling pathway was activated in response to DENV2 infection, leading to suppressed translation of mosquito proteins. In the meantime, PERK inhibition in this study was further shown to increase MMP changes and ROS accumulation, indicating that DENV2-induced ER stress may be reduced by activating the PERK signal pathway since such cellular responses were not evidently shown in uninfected PERKi-treated cells (Figures S1–S3). It is known that GSK2606414 is a small molecule that inhibits PERK phosphorylation [27]. Thus, it may reduce eIF2α phosphorylation [29], resulting in enhanced efficiency of protein translation and the increase of ER stress levels in DENV2-infected C6/36 cells. In addition to ER stress, initiator and effector caspases and the apoptosis rate also obviously increased in DENV2-infected C6/36 cells after the PERK signal pathway was inhibited. This revealed that DENV2-induced ER stress in mosquito cells can be alleviated via the shutdown of cellular proteins by eIF2α, which was phosphorylated by the PERK signal pathway. Undoubtedly, this pro-survival “byproduct” of mosquito cells was also advantageous for viral replication. Indeed, a similar feature was also observed infected by DENV [43].

Flaviviruses are required to compete for the machinery of a host cell during replication due to the lack of an intrinsic translational apparatus [44]. Hypothetically, viral RNAs retain their translation efficiency by utilizing ribosomes released from host mRNAs [9]. This feature ensures maximal viral replication and the possibility of evading a host’s defenses [12,45]. However, it raises an interesting question of how the virus translates its proteins for continuous replication. Although DENVs possess an m7GpppN cap structure at the 5′-end of genomic RNA, they lack a poly A-tail at the 3′-end [46]. As a result, DENVs may utilize a unique mode for protein translation, likely a cap-independent process [26,47]. Cyclization between conserved complementary sequences in both terminal regions of RNA was reported to be the way that proteins are translated in DENVs [48].

A panel of host genes involved in antioxidant defense and/or anti-apoptotic effects was demonstrated to be upregulated in mosquito cells with DENV2 infection [12,22]. As global proteins of infected cells are usually shut down after infection by DENV2, pro-survival genes may be selectively upregulated. This suggests that there might be second machinery in addition to the mode of cap-dependent translation. Many genes involved in antioxidant defense actually possess internal ribosome entry site (IRES)-containing mRNAs, which may be utilized for an alternative mode of protein translation [37,49]. The ER-stress marker, BiP/GRP78, is known to be the first cellular mRNA with a mode of translation by an IRES element in its 5′-UTR [50]. BiP/GRP78 is significantly upregulated in response to DENV2 infection in C6/36 cells [8]; this is eventually beneficial for cell survival under stress induced by virus infection [51]. As no change in translation of this gene was seen in cells treated with the PERK inhibitor [52], we presumed that this gene may shift its translation from a cap-dependent to a cap-independent mode. Apparently, translation of only mRNAs with the structure for canonical cap-dependent protein translation was reduced in mosquito cells with DENV2 infection.

## 5. Conclusions

**Figure 10:**
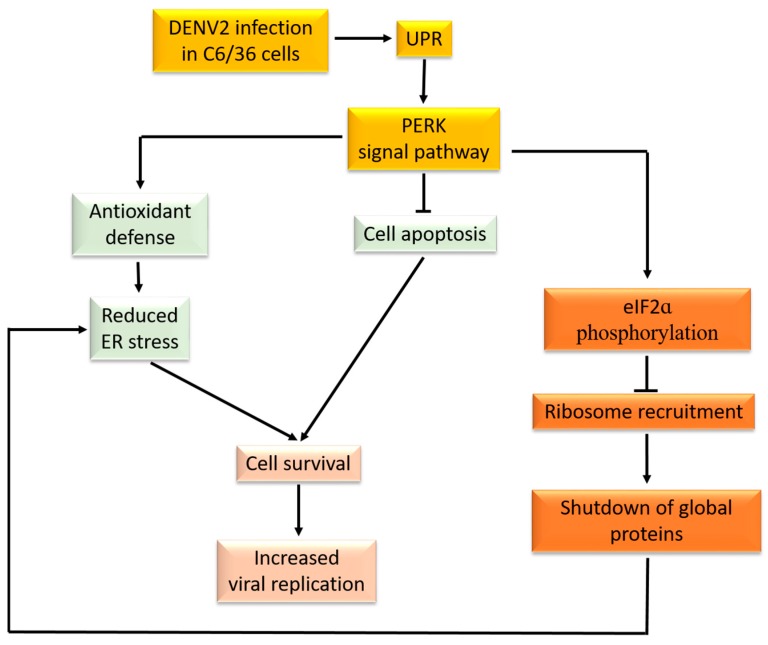
A schematic model of dengue 2 virus (DENV2) infection in mosquito cells, showing the PERK-mediated modulating web involved in dynamic protein synthesis, cell survival and virus replication in cells derived from the mosquito vector. Virus-induced unfolded protein response (UPR) generally activates the PERK signal pathway. Subsequently, global cellular proteins are shut down via impairment of the recruitment of ribosomes binding to the mRNA 5′-cap structure. In the meantime, reduction of endoplasmic reticular (ER) stress helps infected cells survive continuous amplification of the virus as a result of activating antioxidant defense and anti-apoptotic effects. Yellow: virus infection initiates the UPR and its downstream pathway; Light green: Cellular response to the virus-induced UPR; Light red: Cell fate in response to virus infection; Orange red: Protein translation regulation by the virus infection.

DENV2 virus infection in mosquito cells causes a regulatory cascade of protein translation (Figure 10). Specifically, infection causes the UPR that activates the PERK signal pathway. Global cellular proteins are thus shut down via impairment of the recruitment of ribosomes that bind to the mRNA 5′-cap structure. In the meantime, the accumulation of ER stress is reduced, helping infected cells survive continuous amplification of the virus by activating antioxidant defenses and anti-apoptotic effects. This novel finding provides insights into elucidating the PERK-mediated modulating web involved in dynamic protein synthesis, cell survival and virus replication in cells derived from the mosquito vector. This phenomenon is relatively important for elucidating how the mosquito vector can healthily serve as a transmitter of DENV and may-be other arboviruses, as well.

## Supplementary Materials

The following are available online at www.mdpi.com/1999-4915/9/9/262/s1.
