# Supplementary material for: PERK Signal-Modulated Protein Translation Promotes the Survivability of Dengue 2 Virus-Infected Mosquito Cells and Extends Viral Replication
Source: Viruses. 2017 Sep 20;9(9):262. doi: 10.3390/v9090262 (PMC5618028; doi:10.3390/v9090262)
Supplement: Supplementary file 1 [file viruses-09-00262-s001.pdf]

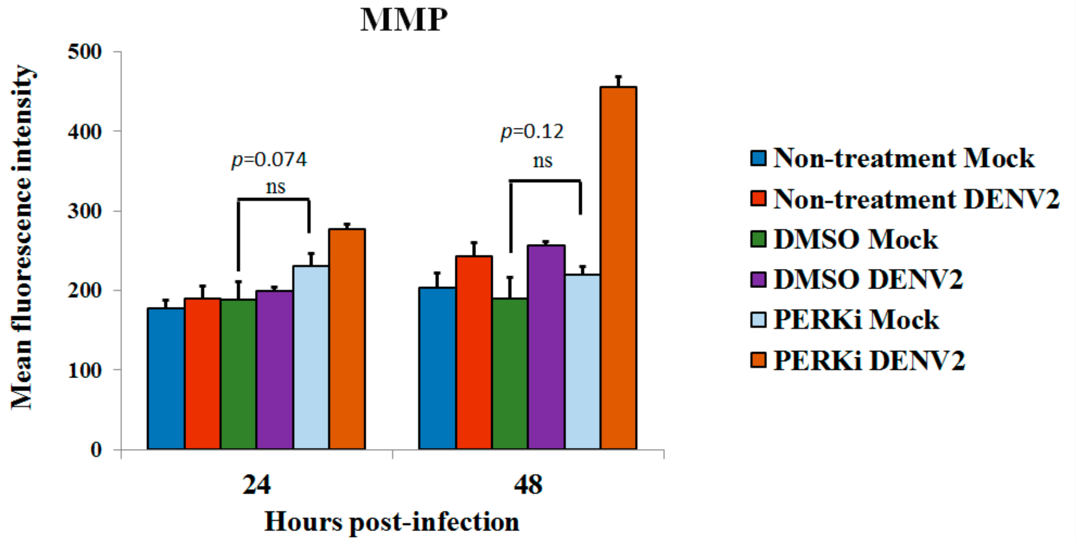

Supplementary Fig. 1. Evaluation of endoplasmic reticular (ER) stress by measuring the mitochondrial membrane potential (MMP) in C6/36 cells with or without infection by dengue 2 virus (DENV2). C6/36 cells were infected with DENV2 at MOI of 1, treated with 5 μM PERK inhibitor (PERKi; GSK2606414) or DMSO, and then incubated for 24 or 48 h. To measure MMP, the FITC channel for green MitoCapture monomers was detected by FACScan flow cytometry. The results revealed that PERKi treatment did not change the MMP in uninfected cells at the time parallel to the time for infection, *i.e.*, 24 and 48 h (Student's *t*-test; ns, no significant difference). Nevertheless, the MMP increased significantly in the DENV2-infected cell with PERKi treatment, particularly at 48 hpi.

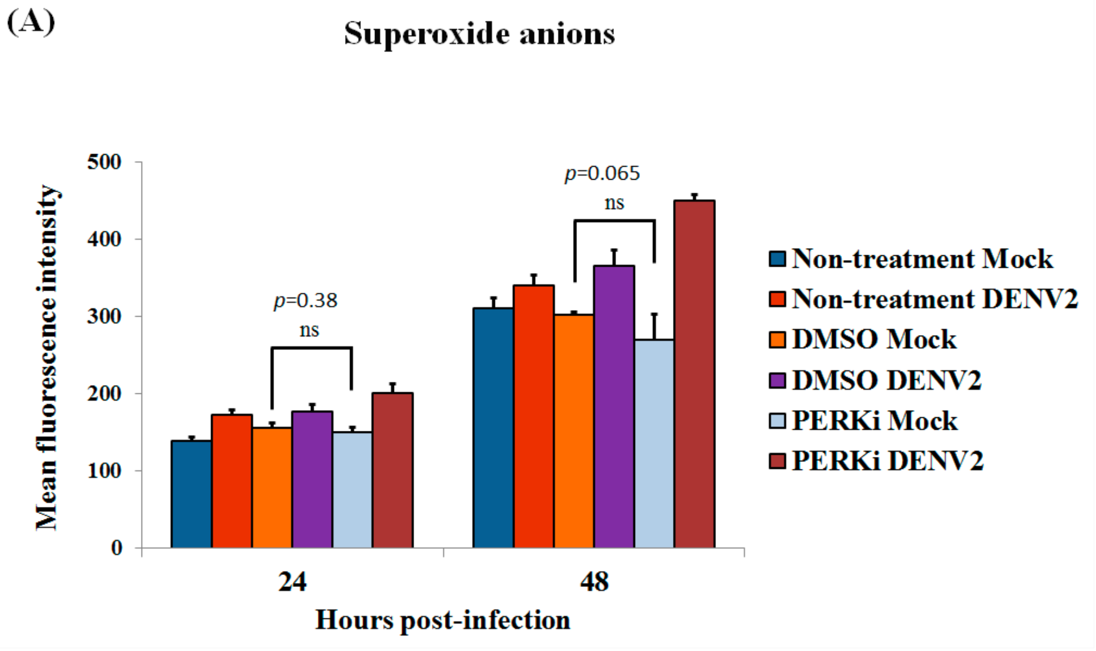

(B)

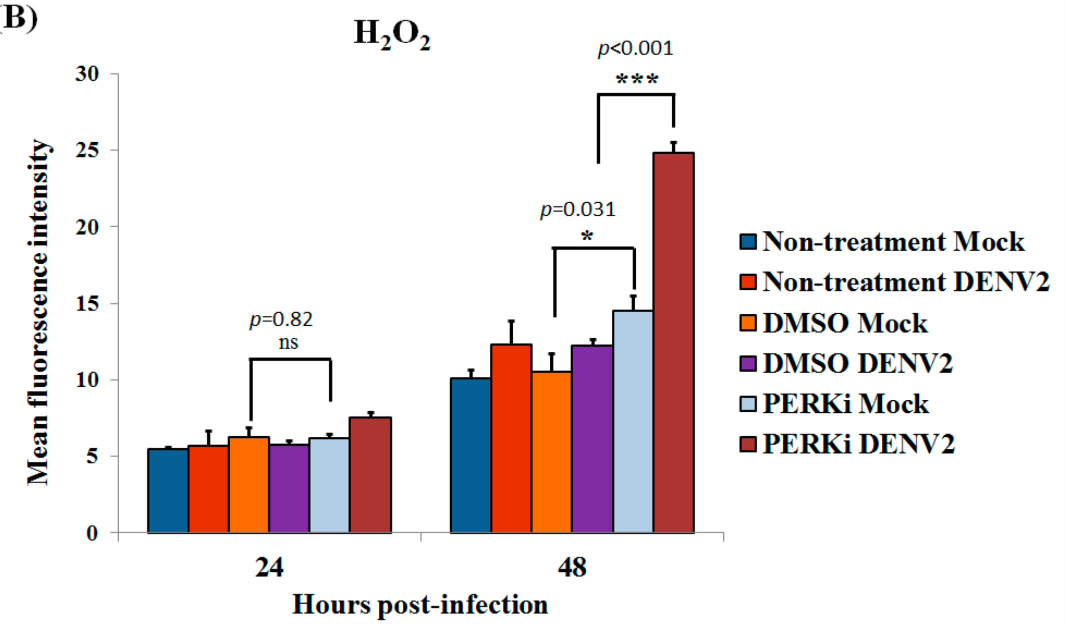

Supplementary Fig. 2. Involvement of the PERK signaling pathway in generating accumulation of reactive oxygen species (ROS) in C6/36 cells with dengue 2 virus (DENV2) infection. C6/36 cells were infected with DENV2 at MOI of 1, treated with 5 μM PERK inhibitor (PERKi; GSK2606414) or DMSO, and then incubated for 24 or 48 h. The cells stained with 10 μM Dihydroethidium were subject to detection of superoxide anions while that with 10 μM H2DCFDA were for detection of H_2O_2 by measuring the fluorescence intensity using flow cytometry. (A) The result showed that PERKi treatment did not affect accumulation of superoxide anions at either time point of treatment, 24 or 48 h. In the meantime, the accumulation level of superoxide anions significantly increased in the DENV2-infected cells with PERKi treatment at 48 hpi. (B) The levels of H_2O_2 accumulation were not shown to change at 24 hpi in uninfected cells either treated with PERKi or not (Student’s *t*-test; no significant difference). Although H_2O_2 accumulation was shown to increase at 48h in uninfected PERKi-treated cells (Student’s *t*-test; $p < 0.05$), it eventually increased at a significant level of difference DENV2-infected PERKi-treated cells (Student’s *t*-test; $p < 0.001$).

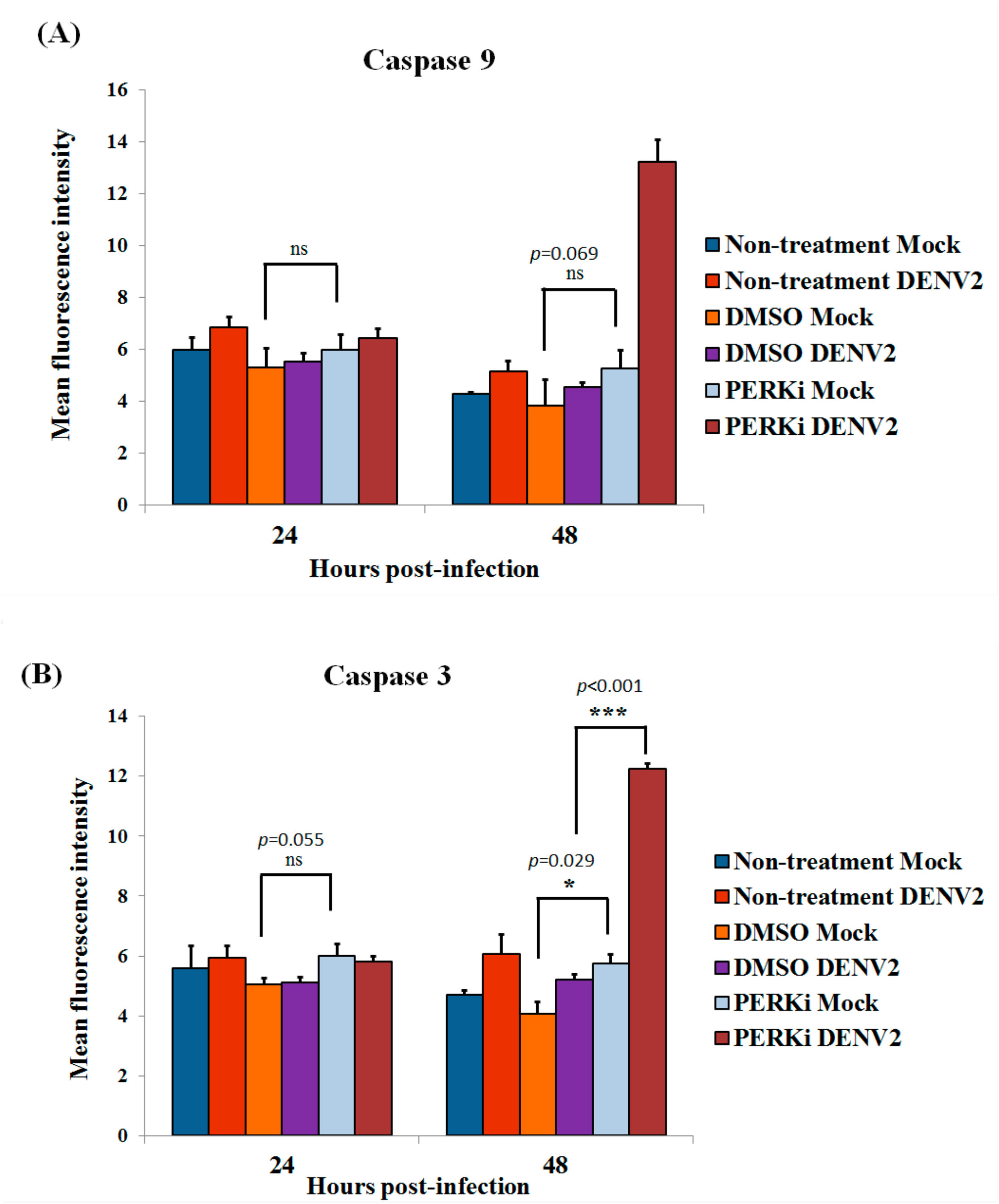

Supplementary Fig. 3. The effect of the PERK inhibitor on activation of caspases-9 (the initiator) and -3 (the effector) in C6/36 cells with dengue 2 virus (DENV2) infection. C6/36 cells were infected with DENV2 at MOI of 1, treated with 5 μM PERK inhibitor (PERKi; GSK2606414) or DMSO, and then incubated for 24 or 48 h. The caspase activities were measured by detecting the fluorescence intensity with flow cytometry, either stained with FITC-LEHD-FMK and FITC-DEVE-FMK for caspase-9 and caspase-3, separately. (A) No evident change of caspase-9 was detected in uninfected cells at 24 h, even they have been treated with a PERKi. In contrast, there was a significant increase in PERKi-treated cells with DENV2-infection of 48 h. (B) Despite caspase-3 activity was shown to increase in uninfected PERKi-treated cells (Student's *t*-test; $p < 0.05$), extremely significant increase was observed

in PERKi-treated cells with DENV2 infection for 48 h (Student's t-test; $p < 0.001$).
